# Pharmacist and Physician Interpretation of Abbreviations for Acetaminophen Intended for Use in a Consumer Icon

**DOI:** 10.3390/pharmacy3040169

**Published:** 2015-10-15

**Authors:** Saul Shiffman, Helene Cotton, Christina Jessurun, Mark A. Sembower, Steve Pype, Jerry Phillips

**Affiliations:** 1 Pinney Associates, 201 N. Craig Street (Suite 320), Pittsburgh, PA 15213, USA; E-Mails: msembower@pinneyassociates.com (M.S.); sype@pinneyassociates.com (S.P.); 2 Department of Psychology, University of Pittsburgh, Pittsburgh, PA 15260, USA; 3 Independent market research consultant, Glenview, IL 60026, USA; E-Mail: hcotton@mac.com; 4 Johnson & Johnson Consumer Inc., Skillman, NJ 08558, USA; E-Mail: CJessuru@its.jnj.com; 5 Drug Safety Institute, Miami, FL 33131, USA; E-Mail: jphillips@brandinstitute.com

**Keywords:** Acetaminophen, icon, abbreviations, medication errors

## Abstract

Concomitant use of multiple acetaminophen medications is associated with overdose. To help patients identify acetaminophen medications and thus avoid concomitant use, an icon with an abbreviation for “acetaminophen” has been proposed for all acetaminophen medications. This study assessed pharmacists’ and physicians’ use and interpretation of abbreviations for “acetaminophen”, to identify abbreviations with other meanings that might cause confusion. Physicians (n = 150) reported use and interpretation of candidate abbreviations Ac and Acm. Pharmacists (n = 150) interpretations of prescription orders using the candidate abbreviations APAP, Ac, Ace and Acm in typed, handwritten or spoken form, were judged for critical confusions likely to cause patient harm. Critical confusion was rare, except for omission by pharmacists of the acetaminophen dose for Hydrocodone/APAP prescriptions (10%). Ac was in common use to indicate “before meals”, and was interpreted as such, but some physicians (8%) said they use Ac to indicate anticoagulant drugs. Most pharmacists (54%) interpreted Ace as acetaminophen, and none interpreted it as referring to ACE-inhibitors. Acm was rarely used in prescriptions, had no common interfering meanings, and was often (63%) interpreted as acetaminophen, especially when prescribed in combination with an opiate (85%). The data validated concerns about abbreviations in prescribing: all abbreviations resulted in some misinterpretations. However, Acm was rarely misinterpreted, was readily associated with “acetaminophen”, and seemed appropriate for use in a graphic icon to help consumers/patients identify acetaminophen medications.

## 1. Introduction

Acetaminophen is an analgesic and anti-pyretic in widespread use in the US, where it is estimated that 20% of adults use acetaminophen in any given week [1]. Excess intake of acetaminophen has been associated with liver injury [2], and with many emergency room visits [3]. Acetaminophen is present in both OTC and prescription medicines, often in combination with other active ingredients. Patients often do not know that acetaminophen is an active ingredient in medicines they are taking [4]. A recent study found that poor knowledge of acetaminophen as an active ingredient in medicines was associated with exceeding the recommended maximum daily dose of 4 grams [5], and that concomitant use of multiple acetaminophen medicines was also associated with overdose [5].

**Figure 1:**
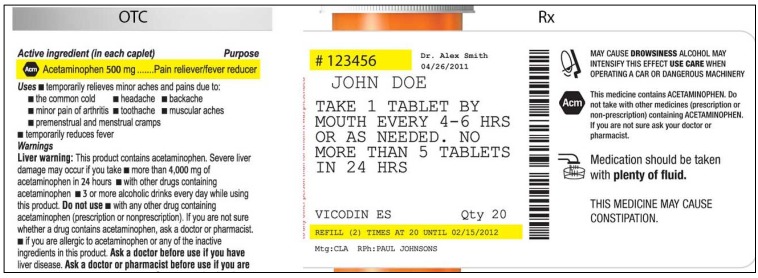
Sample icon-bearing Over the Counter (OTC) and Prescription (Rx) labels.

As a way to help users recognize which medications contain acetaminophen and avoid concomitant use, it has been suggested that an icon be placed on all acetaminophen-containing medicine containers [6]. Consistent with guidance for the design of warnings [7], icon candidates were developed through a process of iterative design, consumer feedback, and qualitative testing. The icon candidates contained letters or an abstract symbol enclosed in a hexagon (see Figure 1 for examples of OTC and prescription labels). In qualitative testing, consumers indicated that the hexagon suggested a stop sign and thus signaled caution, which was deemed an appropriate message. Consumers also indicated that abbreviations suggesting “acetaminophen” helped link the icon to the active ingredient.

Quantitative research with consumers, the target for the icon, showed that APAP performed poorly as a symbol for acetaminophen, because consumers did not see how it was related to “acetaminophen”. In contrast, icons that used letters that abbreviated of the word “acetaminophen” (Ac, Ace, Acm) effectively communicated that acetaminophen was an ingredient [8]. “Ace” was less favored, because of concern it might have promotional connotations, but Ac and Acm were regarded as particularly promising candidates.

The acetaminophen icon is intended as a communication to patients/consumers, and not for health professionals. Nevertheless, it seemed relevant to examine how it might affect prescriber and pharmacist practices if the letters incorporated in the icons were to come into use as a shorthand for “acetaminophen”, much as “APAP” is already used to stand for acetaminophen. The adoption of such abbreviations is discouraged [9], but could nevertheless occur, and could potentially lead to medication errors, particularly if the abbreviations are already being used for other meanings in prescribing. For example, if AC were already being used to designate anticoagulants, or to mean before meals (ante cebum, in Latin), its use to mean “acetaminophen” could lead to medication errors that could potentially harm patients, which are designated as “critical confusions [7]”. Accordingly, the study assessed how often and for what purposes Ac and Acm were already in use in prescribing.

Regardless of current use, it also seemed important to assess how prescribers and pharmacists might interpret the candidate letter combinations if they encountered them on a prescribing order. To assess this among pharmacists, we used the methods commonly used to test new proprietary drug names for confusion [10]: we presented pharmacists with prescriptions using the letters and asked them to interpret them, coding the result for correctness and also for critical confusion. The prescriptions were presented in one of three forms—typewritten, handwritten, or spoken—and both with or without “hydrocodone”, the most common co-ingredient of acetaminophen in prescription medications. Pharmacists’ interpretations were re-tested after the intended meaning of the icons and abbreviations was explained, mirroring the information that would accompany any introduction of the icons. Among physicians, current use of Ac and Acm in prescribing was assessed, along with their interpretation of these letters in prescribing orders.

## 2. Experimental Section

### 2.1. Methods

#### Participants

Pharmacists: A total of 150 pharmacists, representing community/retail (n = 93) and hospital (n = 57) practices, participated in the study. (There were no significant differences in outcomes between retail-and hospital-based pharmacists, so this distinction is not discussed further). A total of 1150 invitations were sent to pharmacists from a panel of 3226 pharmacists maintained by Brand Institute for research on medication names. To participate, pharmacists had to be licensed and practicing/dispensing at least 25% time. Data were collected in two waves, with 75 pharmacists in each (this sample size is typically used to evaluate pharmacist interpretations of proprietary drug names for potential confusion, for FDA consideration). The two waves assessed different sets of letters. The first wave assessed Ac, Ace, and APAP (as well as assessing prescriptions written for Pepcid AC). The second wave assessed Acm and APAP.

Physicians: A total of 1178 invitations were sent to physicians in a Brand Institute database of 9351 physicians, we enrolled 150 physicians engaged at least 50% time in general practice, family practice, or internal medicine in a clinical setting, with responsibility for prescribing. These specialties were targeted as the most common prescribers of acetaminophen medications. Participants were studied in two waves: the first wave was asked about Ac and the second wave about Acm.

Individuals who had participated in research in the previous 3 months and those employed by a pharmaceutical company were excluded. Table 1 shows the characteristics of the resulting pharmacist and physician samples. There were no differences between waves in pharmacists’ or physicians’ demographics.

**Table 1:** Demographics.

Pharmacists			Physicians	
	*N = 150*			*N = 150*
*Gender*			*Gender*	
Female	38.7%		Female	23.3%
Male	61.3%		Male	76.7%
*Practice Environment*			*Practice Environment*	
Hospital pharmacy	38.0%		Hospital/clinic	22.7%
Retail/community pharmacy	62.0%		Managed care/health network	5.3%
			Nursing home/long term care	0.7%
			Private practice	62.7%
			University hospital	8.7%
*Years in Practice*			*Years in Practice*	
Less than 2	4.0%		Less than 2	1.3%
2–10	29.3%		2–10	27.3%
11–20	33.3%		11–20	40.7%
21–30	24.7%		21–30	26.7%
More than 30	8.7%		More than 30	4.0%
*Work Schedule*			*Work Schedule*	
Full-Time	92.0%		Full-Time	97.3%
Part-Time	8.0%		Part-Time	2.7%
*Proportion of Time Spent in Dispensing Environment*			*Proportion of Time Spent in Clinical Environment*	
0%–9%	0.0%		0%–9%	0.0%
10%–24%	0.0%		10%–24%	0.0%
25%–49%	5.3%		25%–49%	0.0%
50%–74%	21.3%		50%–74%	12.0%
75%–100%	73.3%		75%–100%	88.0%

### 2.2. Procedures

Pharmacists: Data were collected via the web. Pharmacists were randomized (n = 50 each) to be presented with prescriptions either in typed text, handwritten text, or spoken text. The prescriptions were always presented in groups of three, with two other prescriptions for existing marketed medications serving as distractors from the relevant stimuli. Table 2 shows examples. Each prescription was presented separately when shown typewritten or handwritten; when spoken, all three prescriptions were presented (via earphones) together, as part of one order. Pharmacists responded in open-ended text boxes indicating their interpretation of product name and, separately, the signatura. Multiple responses were permitted, and “don’t know” was explicitly allowed as a potential response. Respondents were also asked to state what they would do if they received such a prescription order, and their responses were coded, particularly to capture indications they would dispense a medication.

**Table 2:** Sample presentations of handwritten and typed prescriptions for target stimuli and distractors.

Stimulus	Typed prescriptions	Handwritten prescriptions
Target Stimulus: ACM 325mg 2 po Q4hr #20	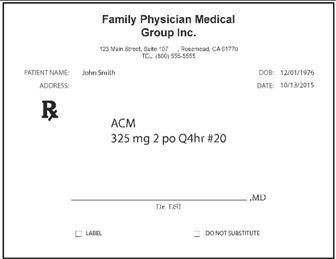	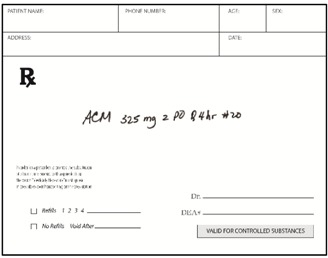
Target stimulus: Hydrocodone 5 mg/ACE 500mg, 1 po every 4–6 hours prn #30	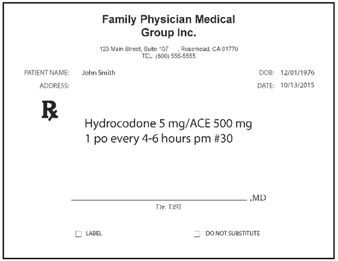	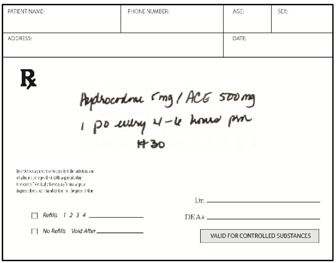
Distractor: Amaryl 2 mg PO QD #30	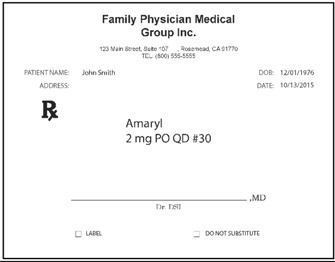	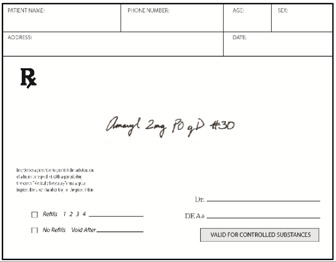

In Wave 1 pharmacists responded to three relevant prescriptions. Two were drawn randomly from Ac, Ace, and APAP; the third was always Pepcid AC. In Wave 2, pharmacists responded to two prescriptions, for Acm and APAP (in randomized order). In both waves, icon letter combinations were randomly rotated through two presentations, one that also showed the co-ingredient “hydrocodone” and one that did not. Thus, in both waves, each pharmacist saw and interpreted 2 acetaminophen-relevant prescriptions, along with several distractors. After initially responding to the test prescriptions without any information about the intended meaning of the abbreviations, pharmacists were exposed to brief text explaining their intended use as icons to mark acetaminophen medications using Ac (Wave 1) or Acm (Wave 2) as examples. (Copies of the material are available from the authors.) After this orientation, pharmacists were retested on stimulus prescriptions containing these letters.

Before being oriented to information about the icon, pharmacists were asked how often they encountered Ac (Wave 1) or Acm (Wave 2) on prescriptions, and how they interpreted these letter combinations.

Physicians: Physicians evaluated Ac in Wave 1 and Acm in Wave 2, being asked if they ever used the candidate letters in prescribing, and, if so, how often and for what purpose. In Wave 1 only, physicians were further asked, as a closed-ended yes/no question, whether they ever used Ac to stand for “anticoagulant”. All physicians were subsequently asked how they would interpret the letters if they encountered them on the prescribing orders of a patient’s chart or medical record, writing text responses in an open-ended text field. Multiple responses were permitted.

### 2.3. Measurements

Qualitative coding: Pharmacists’ open-ended text interpretations of prescriptions were thematically coded by two coders, noting when the prescription was interpreted as “acetaminophen”; other interpretations (including other medications) were tallied. Additional items were also coded thematically. Percent agreement ranged from 94% to 100% across items, averaging 97%.

Adjudicated critical confusion: Three expert judges, blind to stimulus and condition, coded the pharmacists’ and physicians’ interpretations to identify the presence of critical confusion, defined as interpretation and behavior that was likely to expose the consumer to clinical harm - We are grateful to Louis Cantilena, MD, PhD, Jan Engle, PharmD, and Randy Juhl, PhD—all former members or chairs of the FDA’s Nonprescription Drugs Advisory Committee) for their contribution in making these judgments). Pair-wise agreement among the expert judges averaged 94% for the pharmacist responses and 95% for the physician responses. Critical confusion was considered to have occurred whenever any two judges so indicated. A single judge indicated critical confusion in 8% of pharmacist responses and 5% of physician responses; this was almost all due to a particular judge assigning critical confusion to situations that the other two judges did not (e.g., failing to list the acetaminophen content of an opiate-acetaminophen medication).

### 2.4. Analysis

We tallied the percentage of responses considered to be critical confusion, by icon, for pharmacists and physicians. In the pharmacist data, we analyzed how often each icon was interpreted as acetaminophen, when presented with or without hydrocodone as a co-ingredient, and when presented in typed, handwritten, and spoken form. Because the comparisons involved a mix of within-subjects and between-subjects contrasts, the analysis used Generalized Estimating Equation methods [11], a form of regression analysis that accommodates this for dichotomous end-points. We also assessed whether there were any differences in response between retail- and hospital-based pharmacists. Statistical tests used a p-value of 0.05.

## 3. Results

### 3.1. Current Use and Interpretation of Ac and Acm

Pharmacists: Most (81%) pharmacists reported that they had seen the letters Ac used on prescriptions, with most of those (61%) indicating that it appeared on no more than 10% of prescriptions. Three quarters (75%) of pharmacists indicated that they would interpret Ac as “before meals” (or similar meal-related constructs), and 17% would interpret it as “acid control”. Another 9% interpreted Ac as “acetaminophen”. A small number of pharmacists interpreted it as other medications (Pepcid AC, 3%; codeine, 3%, ACE inhibitor, 1%; anti-coagulant, 1%). The remainder gave other, less relevant responses, or said they did not know.

In contrast, only 7% of pharmacists reported that they had ever seen the letters Acm on prescriptions. A total of 63% indicated that they would interpret it as acetaminophen. An additional 13% interpreted Acm as referring to a meal-related instruction. There were single instances of interpretations as other drugs (one each as Alclometasone, Actoplus Met, aspirin, anti-cholinergic medication, and the three-drug combination adriamycin/cyclophosphamide/methotrexate). A total of 27% provided non-drug responses, or said they did not know.

Physicians: A total of 65% of physicians said they use the letters Ac in their current prescription writing practices; 90% of these used it to refer to before meals or with meals. (The others gave non-drug responses or no response). In this open-ended query, none of the physicians said they used Ac to refer to anticoagulants. However, when specifically asked a yes/no question whether they use Ac to mean anticoagulant, 8% of the physicians indicated they did.

In contrast, only 20% of physicians said they use the letters Acm in prescriptions. Among those, reference to “before meals” and “acetaminophen” were the most common uses given (25% and 17%, respectively). Idiosyncratic responses given by one physician each included medication classes such as anticholinergic medication and anticonvulsive medication, and the three-drug combination adriamycin, cyclophosphamide, methotrexate. Other interpretations did not relate to medications.

### 3.2. Critical Confusion

Pharmacists: Adjudicators identified 11 pharmacist interpretations they considered to be critical confusions (*i.e*., deemed likely to harm patients). Critical confusion was most commonly seen in interpretations of APAP: in 6 instances (of 62; 9.7%), pharmacists were presented with a prescription order for “Hydrocodone APAP, 5 mg/500 mg”, but either failed to note the presence of acetaminophen or failed to note the acetaminophen dose, which judges considered to be critical confusion. An additional 4 pharmacists (of 75; 5.3%), presented with prescriptions for “Hydrocodone AC 5 mg/500mg”, failed to clearly note the acetaminophen dosage. One pharmacist did not interpret Ace but simply repeated it in the response, and this was judged to be a critical confusion. No critical confusions were noted for Acm or for Pepcid AC.

Physicians: Physicians asked to interpret an entry for Ac on the prescription order page of a patient’s chart or medical record made no critical confusions. Among interpretations of Acm on the prescription orders, critical confusions were judged when three physicians interpreted the orders as medical diagnoses rather than medications: one as “acute cardiomyopathy” and two as “alcoholic cardiomyopathy”.

### 3.3. Interpretation of Abbreviations 

Pharmacists: When abbreviations were not interpreted as acetaminophen (see below), respondents often simply repeated the abbreviation without interpreting it. No respondent interpreted Ace as “ACE inhibitor”, and none interpreted Ac as “anti-coagulant”. Pepcid AC was almost never (0.7%, 1 response) mistaken for acetaminophen, whether before or after the Ac icon for acetaminophen was explained. Altogether, among the 750 responses to hypothetical prescriptions, there was only one instance in which a pharmacist indicated he/she would dispense another drug, without checking with the prescriber—a pharmacist who interpreted Ac as aspirin and was prepared to dispense it.

As expected, given that it is an established abbreviation for “acetaminophen”, APAP was fairly consistently identified as acetaminophen, whether hydrocodone was mentioned or not (Table 3). The other letter combinations have not been established as abbreviations for acetaminophen, and thus were not expected to be interpreted as such, and generally were not so interpreted, with rates lower than that for APAP. Interpretation as acetaminophen rose significantly when hydrocodone was also mentioned in the order (*p* < 0.0001). In the case of Acm/hydrocodone, this rose to 85% recognition, which approached the figure for APAP on its own (difference, *p* > 0.18).

**Table 3:** Pharmacist interpretation of candidate abbreviations as acetaminophen.

	Ac^1^		Acm^1^		Ace		APAP^2^		
	*Alone*	*Opiate*	*Alone*	*Opiate*	*Alone*	*Opiate*	*Alone*	*Opiate*	*p-value* *Across conditions*
*Pre Orientation*									
All	14.8%^a^	56.0%^b^	28.6%^a^	85.0%^c,d^	32.0%^a^	78.3%^b,c^	93.7%^d,e^	98.4%^e^	<0.0001
Overall	34.6%^a^		58.7%^b^		54.2%^b^		96.0%^c^		<0.0001
*Post Orientation*									
All	72.0%*	80.0%	78.7%*	81.3%					ns
Overall	76.0%*		80.0%*						ns

Different superscript letters indicate significant differences (*p* < 0.05). * Change from pre- to post-orientation, *p* < 0.001; **^1^** Only Ac and Acm were re-tested after orientation; **^2^** Combines data from Round 1 and Round 2.

Not surprisingly, interpretation of Ac and Acm as “acetaminophen” rose significantly after the intended meaning of the icon was explained (Table 3; *p* < 0.0001). These increases were more modest for prescriptions that also mentioned hydrocodone, as these were already often interpreted as acetaminophen, even before the explanation. This was especially so for Acm/hydrocodone, which was as likely to be interpreted as acetaminophen without explanation as with it.

Interpretation of letters was marginally affected by the mode of presentation (*p* < 0.06). Spoken orders for Ac, Ace, and Acm were less often interpreted as acetaminophen than typed or handwritten orders (36.7% versus 54.4% for typed, and 60.3% for handwritten, *p* < 0.02). A similar pattern was seen for APAP (90% vs 100% and 97.6%, *p* < 0.05).

Physicians: When asked how they would interpret Ac if they saw it on a prescription order sheet in a patient’s medical record (Wave 1), most physicians (75%) indicated they would interpret it as an instruction about meals, while 10% said they did not know or indicated no response. Interpretation as “acetaminophen” was rare (n = 1, 1.25%). One physician interpreted Ac as “anticoagulant” and one as “with codeine”; there were no other drug-related responses.

When interpreting Acm, when written in other physicians’ prescribing orders (Wave 2), most physicians (61%) responded “don’t know”. Most of the remaining responses did not refer to medications or prescribing directions. Among those that did, “before meals” (11%) and acetaminophen (9%) were the most common. Some responses referred to medication classes (anticholinergic, 3%, anticonvulsants, 1%) rather than specific medications, and isolated responses referred to medication combinations (1 each albumin, calcium, magnesium and adriamycin, cyclophosphamide, methotrexate).

## 4. Discussion

These studies were part of a program evaluating icons that could help consumers/patients taking OTC and Rx medications recognize products containing acetaminophen. The icon is intended as a communication to the patient/consumer, and its letters are not intended for use on prescription orders, where abbreviations are discouraged [9]. Nevertheless, it was considered prudent to evaluate possible issues that could arise from use of these abbreviations in prescribing.

The study confirmed that APAP is already a well-established shorthand for acetaminophen among physicians and pharmacists. However, it resulted in the highest incidence of critical confusion, as interpreted by the expert judges, in pharmacists’ interpretations of prescribing orders, primarily because pharmacists failed to note the acetaminophen dose in their interpretation. Indeed, problems with APAP are well known—it is on the Institute of Safe Medication Practices’ List of Error-Prone Abbreviations that “should NEVER be used [12]”. Further, APAP tested very poorly with consumers [8], the primary target audience, who did not understand how the letters could relate to “acetaminophen”. This makes APAP unsuitable as the basis for a consumer-directed acetaminophen icon.

While Ac tested well with consumers, the present study showed that these letters are in widespread use to mean “before meals”. Prescribers reported using it that way and pharmacists reported interpreting it that way. Additionally, some prescribers said they were using it to refer to anticoagulants. (Notably, patients taking anticoagulants showed no such confusion [8]). Reinforcing the conclusion that Ac is already in use for other meanings, a search of Davis’ on-line Dictionary of Medical Abbreviations [13] found 33 cited meanings for Ac, which included “before meals”, but not “acetaminophen”. Thus, Ac seems a less suitable letter combination for an acetaminophen icon.

The study did not elicit competing interpretations of Ace: No pharmacist in the study interpreted Ace as “ACE inhibitor”. However, searching the Medical Abbreviations dictionary [13] turns up multiple competing meanings, including several related to ACE inhibitors, suggesting Ace might not be an ideal letter set for an acetaminophen icon.

In contrast to some of the other letter combinations, Acm was not currently in common use. Few pharmacists reported seeing it used on prescriptions, and among the fraction of physicians who reported using it, it was already used to stand for acetaminophen. Although individual physicians and pharmacists occasionally interpreted Acm to refer to drug combinations, such interpretations seemed idiosyncratic, and it seems highly unlikely that such combinations would be administered to a patient based on a prescription for acetaminophen. Similarly, a few physicians said they might interpret Acm as indicating a diagnosis, even when it appeared on a prescribing order, not elsewhere in a patient’s chart. These interpretations also seemed idiosyncratic: a Medical Abbreviations dictionary [13] search turned up only two established uses, which did not refer to diagnoses, medications, or prescription orders. Absent such competing meanings, Acm seemed to become readily identified with acetaminophen. Indeed, some physicians reported already using it to stand for acetaminophen, and, when the order included the term hydrocodone, Acm was interpreted as “acetaminophen” by 85% of pharmacists, even before the intended meaning was explained. Importantly, since the icon is intended as a communication to consumers, Acm was highly rated by consumers [8], suggesting Acm is a suitable candidate for inclusion in an acetaminophen icon intended for consumers and patients.

Other than the noted critical confusions involving APAP, expert judges saw scant potential for critical confusion arising from use of the studied abbreviations for acetaminophen. Indeed, in 750 dispensing decisions studied, the only instance in which a pharmacist indicated a readiness to dispense an incorrect medication was one instance in which aspirin was to be dispensed to a patient whose prescription called for acetaminophen.

Pharmacists were less likely to interpret prescription orders as acetaminophen when they were communicated orally, consistent with other findings indicating that spoken orders are more error-prone [14]. As the proposed acetaminophen icons are intended as visual cues to consumers and patients and not verbal markers for professionals, they should not be introduced into oral communication.

It is important to emphasize that the development of an icon for acetaminophen directed to consumers is not intended to encourage any use of abbreviations in prescribing. Indeed, the findings reinforce the best-practice recommendation to avoid using abbreviations [9], as even the best-established abbreviation we tested was subject to some misinterpretation. The icons being considered for acetaminophen medications are not intended as short-hand abbreviations for prescribers, but as graphic and iconic communications to patients, on patient medication containers or to consumers on OTC medication labeling, to convey the presence of acetaminophen. Research with consumers showed that the abbreviation-based icons were regarded as effective at communicating that acetaminophen was present, and were not misinterpreted in any way that could be harmful: adjudicators found no instances of critical confusion among the consumers responses [8].

### Limitations

The study had some limitations. Although the study sampled both community and hospital-based pharmacists, it is not known that the sample was representative of all pharmacists. Also, larger samples may have enabled more precise inferences, but we used sample sizes often used to evaluate potential for confusion in prescription orders [10]. We studied participants’ responses to an online test, and not their actual dispensing practices.

The study also had strengths. To evaluate interpretations of prescriptions, we used a test methodology that is used to test new proprietary drug names to identify potential for confusion and medication errors. We engaged experts in internal medicine and pharmacy to judge when misinterpretations of prescriptions were likely to bring harm to patients, providing for expert judgment regarding potential problems.

## 5. Conclusions

Responses from both physicians and pharmacists indicated that Ac might be an unsuitable abbreviation to use inside an acetaminophen icon, because it had interfering meanings already in use among health professionals. In contrast, Acm appeared to be more easily associated with acetaminophen, and was not in common use for other meanings, making it a good candidate to be part of an icon to enable OTC consumers and Rx patients to identify medications containing acetaminophen. If testing shows that it can help consumers make appropriate medication decisions, an icon based on Acm may help avoid medication errors, acetaminophen overdose and acetaminophen toxicity. In any case, the efforts of pharmacists and prescribers will be needed to help educate patients and consumers about proper use of acetaminophen, and about the meaning and utility of an acetaminophen icon.
